# Hernie diaphragmatique post-traumatique de l’enfant: à propos d’un cas au Centre Hospitalier Universitaire Pédiatrique Charles de Gaulle de Ouagadougou

**DOI:** 10.11604/pamj.2013.16.55.2894

**Published:** 2013-10-16

**Authors:** Rawéléguinbasba Armel Flavien Kabore, Emile Bandre, Toussaint Tapsoba, Isso Ouedraogo, Ibrahim Alain Traore, Nazinigouba Ouedraogo, Albert Wandaogo

**Affiliations:** 1 Centre Hospitalier Universitaire Pédiatrique Charles de Gaulle de Ouagadougou, Burkina Faso; 2 Centre Hospitalier Universitaire Souro Sanou de Bobo Dioulasso, Burkina Faso; 3 Centre Hospitalier Universitaire Yalgado Ouédraogo, Burkina Faso

**Keywords:** Hernie diaphragmatique, traumatisme abdominal, enfant, diaphragmatic hernia, abdominal trauma, infant

## Abstract

La hernie diaphragmatique post-traumatique est une urgence chirurgicale rare chez l’enfant mais pouvant mettre rapidement en jeu le pronostic vital. Les auteurs rapportent le cas d’un garçon de 04 ans admis aux urgences pour douleur abdominale suite à une contusion thoraco-abdominale par accident de la voie publique. Le bilan radiologique initial a consisté en une échographie abdominale qui a révélé un hémopéritoine de petite abondance sans lésion focale. Douze heures après son admission, le patient a présenté une détresse respiratoire avec tableau clinique de pneumothorax gauche qui a nécessité une exsufflation en urgence. Le diagnostic de hernie diaphragmatique gauche a été posé à la radiographie du thorax réalisée après la ponction. L’enfant a bénéficié d’une cure chirurgicale. L’évolution a été favorable. La hernie diaphragmatique post traumatique, bien que rare chez l’enfant, devrait être systématiquement recherchée par une radiographie thoracique ou un scanner thoraco-abdominal devant tout traumatisme abdominal avec hyper pression. Son traitement est chirurgical.

## Introduction

La hernie diaphragmatique post traumatique est une pathologie peu fréquente chez l’enfant, compliquant les traumatismes fermés ou pénétrants de l’abdomen et du thorax. Le diagnostic en phase aiguë est difficile et l’incidence élevée des lésions associées font de cette pathologie une urgence médico-chirurgicale avec une mortalité élevée [1–3]. Nous rapportons un cas de hernie diaphragmatique par traumatisme fermé de l’abdomen chez un enfant, diagnostiquée au décours d’une détresse respiratoire.

## Patient et observation

Il s’est agi d’un garçon 4 ans, reçu au Centre Hospitalier Universitaire Pédiatrique Charles de Gaulle de Ouagadougou le 06/07/09 pour contusion abdominale et lésions cutanées multiples suite à un accident de la voie publique survenu 8 heures avant son admission. L’enfant traversant une route a été percuté par une voiture et aurait reçu une roue sur l’abdomen. Ce mécanisme d’hyperpression abdominale s’est accompagné d’une émission de selles puis de douleurs abdominales intenses et diffuses. L’enfant est resté conscient. Son état hémodynamique était stable. Une échographie abdominale a objectivé une lame d’épanchement liquidien intra-péritonéal sans lésion viscérale.

Douze heures après l’admission est survenue une détresse respiratoire majeure avec à l’examen clinique un syndrome d’épanchement gazeux pleural gauche. Une ponction exsufflation pleurale a été réalisée en urgence; elle a ramené de l’air avec des traces de liquide d’aspect verdâtre, ce qui a entraîné une amélioration de l’état respiratoire.

**Figure 1:**
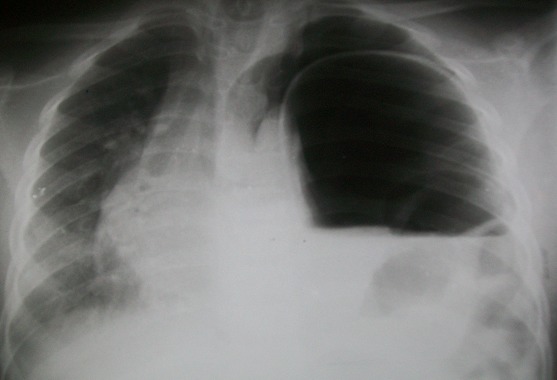
Radiographie thoracique préopératoire

**Figure 2:**
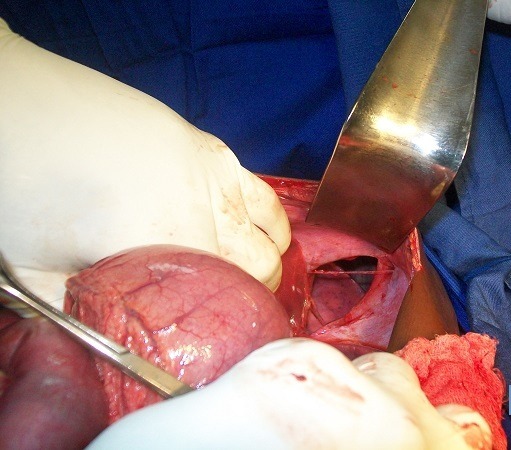
Vue peropératoire

La radiographie du thorax (Figure 1) réalisée au décours de la ponction a montré un effacement de l’hémi-coupole diaphragmatique gauche, une image de clarté digestive occupant la quasi-totalité de l’hémi-thorax gauche avec une déviation du médiastin. Il n’y avait pas de lésion osseuse associée. L’hypothèse d’une hernie diaphragmatique post-traumatique a été évoquée, posant ainsi l’indication opératoire. L’abord chirurgical a été une c’liotomie transversale sus-ombilicale réalisée sous anesthésie générale avec intubation oro-trachéale. L’exploration de la cavité péritonéale a objectivé, une hernie de l’estomac à travers une déchirure diaphragmatique linéaire de 10 cm de long siégeant sur le centre de la coupole gauche (Figure 2). La recherche de lésions associées a permis d’identifier une perforation iléale punctiforme sur le bord anti-mésentérique à 50 cm de la jonction iléo-caecale. Les gestes réalisés étaient une réduction de la hernie, une suture de la brèche diaphragmatique en deux plans en points séparés simples au fil non résorbable et une résection cunéiforme avec suture de la perforation iléale. La réanimation post-opératoire a consisté en une oxygénothérapie au masque facial, une triple antibiothérapie associant la ceftriaxone, la gentamycine et le métronidazole injectables, une perfusion de cristalloïdes, une analgésie avec du paracétamol injectable. Les suites opératoires ont été marquées par une hyperthermie et un syndrome péritonéal à J3 post-opératoire indiquant une nouvelle laparotomie qui s’est révélée blanche. Il a été réalisé un lavage de la cavité péritonéale avant la fermeture. Le patient est sorti après 15 jours d’hospitalisation et est resté asymptomatique après sa sortie. Les contrôles radiographiques à une semaine puis 3 mois sont revenus normaux.

## Discussion

Les traumatismes thoraco-abdominaux peuvent être, bien que rarement, à l’origine d’une hernie diaphragmatique chez l’enfant [1, 4]. Les principales circonstances de survenue sont les accidents de la voie publique [5], grands pourvoyeurs de traumatismes graves.

Le mécanisme prédominant semble être l’hyperpression intra-abdominale, ce qui expliquerait la plus grande fréquence de la hernie diaphragmatique dans les traumatismes fermés: ainsi, Sôzubir et Ramos ont retrouvé dans leurs séries une prédominance des traumatismes fermés avec respectivement 5/8 et 13/15 cas [1, 2]. Ce mécanisme peut aussi rendre compte, avec la moindre implication des enfants dans la traumatologie majeure, de la rareté relative de cette atteinte chez l’enfant dont la structure thoraco-abdominale est plus souple que celle de l’adulte [5]. La coupole diaphragmatique gauche est à priori plus exposée à la rupture du fait de l’absence de protection viscérale contre l’hyperpression intra-abdominale: son atteinte est retrouvée dans notre observation. Cependant, l’atteinte bilatérale est possible [2].

En phase aiguë, l’association variable de signes souvent peu spécifiques peut permettre d’évoquer le diagnostic. Ce sont: des douleurs de la partie supérieure de l’abdomen ou de la base du thorax, une dyspnée, des lésions costales, une fracture du bassin ou du rachis lombaire qui sont le reflet d’une compression majeure du tronc, une matité ou un tympanisme de la base d’un hémi-thorax, des bruits intestinaux dans le thorax [1]. Notre patient ne présentait que des douleurs abdominales au début et ce n’est que secondairement que se sont associés une détresse respiratoire et un syndrome d’épanchement pleural gazeux gauche.

La confirmation du diagnostic est apportée par l’imagerie. La radiographie standard du thorax montre souvent des anomalies diverses (85% des cas dans la série de Sôzûbir), mais seulement un tiers est pathognomonique [1]. Cette radiographie peut être normale dans plus de 50% des cas [4]. Comme dans notre cas, la radiographie thoracique suffit parfois pour poser le diagnostic (53% des cas dans la série de Ramos [4]. L’échographie abdominale est performante en cas de hernie viscérale. Le transit ’so-gastro-duodénal, le scanner et l’imagerie par résonnance magnétique (IRM) permettent un diagnostic plus précis des lésions. [1, 2]

Le pneumothorax constitue le principal diagnostic différentiel et peut conduire à une ponction exsufflation comme dans notre cas, voire un drainage en l’absence de bilan radiologique, ce qui expose à une lésion iatrogène de l’organe hernié [1]. Des lésions viscérales et musculo-squelettiques peuvent s’y associer et aggraver ainsi le pronostic vital [1, 5].

La lésion associée dans notre cas était une perforation du grêle probablement due au choc direct avec hyperpression intra-abdominale. Cette pression était forte au point de vaincre le sphincter anal, provoquant une émission spontanée de selles. Ramos CT et al. ont retrouvé des perforations intestinales associées dans 33% [2].Le traitement de la hernie diaphragmatique post-traumatique est chirurgical. L’abord chirurgical peut être une c’liotomie ou une thoracotomie. La c’liotomie a été pratiquée dans notre cas du fait de la contusion abdominale pour rechercher des lésions viscérales associées. La thoracotomie est indiquée dans les cas de hernies localisée à droite ou dans celles en phase de chronicité [6]. Lorsque le patient est stable, la cure de la hernie peut être réalisée par coeliochirurgie [7, 8] mais la présence de lésions associées comme les perforations intestinales et les lésions du rétropéritoine limite son usage [4]. Bien que dans notre cas, l’évolution ait été favorable, la hernie diaphragmatique est une pathologie létale dans 20-50% des cas [4] du fait de la gravité des lésions associées [5].

## Conclusion

La hernie diaphragmatique post-traumatique chez l’enfant est une pathologie chirurgicale peu fréquente. Sa gravité est surtout liée aux lésions associées pouvant égarer le diagnostic et aussi mettre directement en jeu le pronostic vital du malade. Elle doit donc être recherchée par un bilan radiologique comportant au minimum une radiographie thoracique et une échographie abdominale devant tout traumatisme abdominal avec mécanisme d’hyperpression abdominale.
